# Thoracic Empyema as Rare Complication of an Appendicular Mass: A Case Study and Review of the Literature

**DOI:** 10.1155/2018/9640397

**Published:** 2018-05-15

**Authors:** George Vasquez-Rios, Lesly Calixto-Aguilar, Richard Pajuelo, Wilder Alarcon

**Affiliations:** ^1^Instituto de Medicina Tropical Alexander von Humbolt, Universidad Peruana Cayetano Heredia, Lima, Peru; ^2^Facultad de Medicina Alberto Hurtado, Universidad Peruana Cayetano Heredia, Lima, Peru; ^3^Departamento de Cirugia Pediatrica, Hospital Cayetano Heredia, Lima, Peru; ^4^Departamento de Pediatria, Hospital Cayetano Heredia, Lima, Peru

## Abstract

**Introduction:**

Thoracic empyema is an infrequent complication of appendicitis that has rarely been reported in the literature.

**Case Presentation and Review of the Literature:**

The case of a 11-year-old boy who was admitted for medical management of an appendicular mass is presented. His clinical course was complicated by the development of an appendicular abscess and an extensive right-sided empyema. A comprehensive review of the literature was conducted including the most representative cases. The data were collected and analyzed by two independent investigators. Ten cases were found. Most patients were young individuals (mean age: 25.1 years; male : female ratio: 0.5). Risk factors for thoracic empyema included pregnancy (10%) and age (60%). The most frequent organisms isolated were *Escherichia coli, Bacteroides* spp., and *Klebsiella* spp. The survival rate was 100%.

**Conclusion:**

Thoracic empyema should be considered a potential cause of respiratory distress in patients with appendicitis. Furthermore, the abdomen should be carefully evaluated as a source of infection in patients with thoracic empyema without an underlying lung disease.

## 1. Introduction

Appendicitis is one of the most frequent surgical emergencies in adult and pediatric populations [1, 2]. Common complications include appendicular phlegmon (appendicular mass), appendicular abscess, and peritonitis. Shock, sepsis, or atelectasis in the postoperative period has been associated with respiratory disease in this group of patients [3, 4]. However, empyema has rarely been reported as a complication of appendicitis. We present the case of a young patient with an appendicular mass whose clinical course was complicated with thoracic empyema. In addition, we reviewed the literature on this topic.

## 2. Case Presentation and Review of the Literature

**Figure 1:**
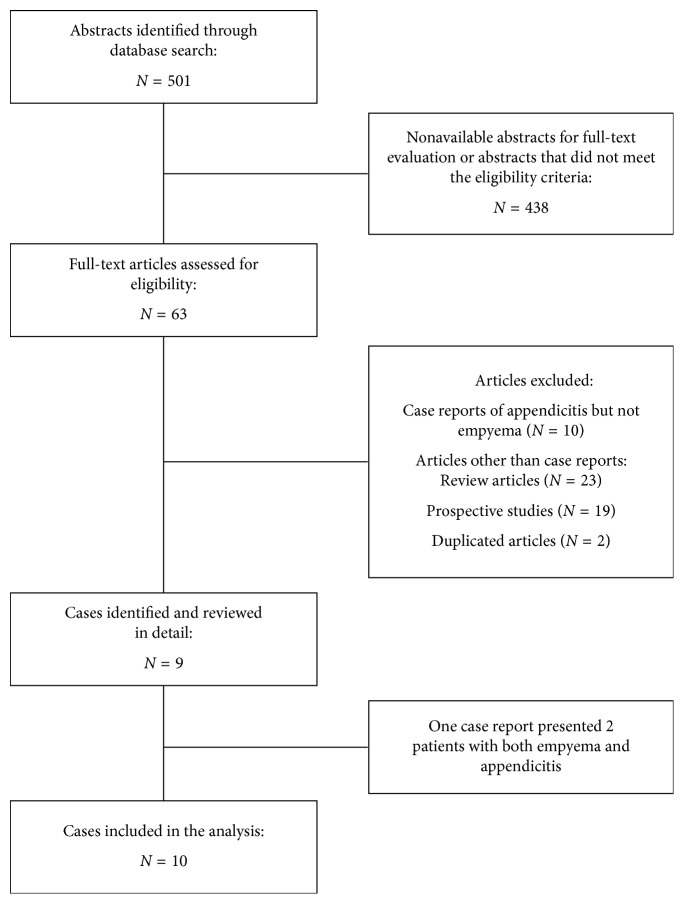
Flowchart of the literature search and articles included in this study.

We searched the medical literature during August 2017 to identify cases of empyema in the setting of appendicitis. Reports presenting patients with thoracic empyema or lung abscess as a complication of appendicitis in the preoperative or postoperative period were eligible. Two independent investigators (GVR and LCA) reviewed abstracts through PubMed, MEDLINE, LILACS, and SciELO in either English or Spanish language. Any discrepancy was solved by consensus among the authors. Search terms included (Mesh) [appendicular mass AND empyema]; [appendicitis AND empyema]; [appendicular abscess AND empyema]; [appendicitis AND respiratory distress]; [appendicitis AND pleural infection] and [appendicitis AND lung abscess]. Those articles meeting the eligibility criteria were reviewed in detail. Additionally, we complemented the literature search by reviewing Google/Google Scholar and previous references. The information was collected in case forms and analyzed with Microsoft Excel 2016.  Figure 1 shows the flowchart of articles screened and included in this study.

### 2.1. Case presentation

An 11-year-old boy presented to the pediatric emergency department with right lower quadrant abdominal pain, vomiting, and fever for the past 4 days. In addition, he endorsed decreased appetite and no bowel movements. His mother mentioned that the patient took ibuprofen 48 hours prior to presenting to our institution. Upon examination, vitals were as follows: BP: 90/60 mmHg, HR: 128 bpm, RR: 28 rpm, *T* :  38.5 C, and SO_2_: 98% on room air. Physical evaluation of the abdomen revealed a tender right lower quadrant with a well-defined, nonindurated mass. No rebound or guarding was noted. The rest of the physical examination was unremarkable.

Blood tests showed a WBC count of 29.7 × 10^3^ cells/mm^3^ (*N*: 81.4% *B*: 0.1% *L*: 2.5% *E*: 1%), platelet count of 370 × 10^3^ cells/mm^3^, Hb of 13.6 g/dL, and Hct of 36%. Biochemical studies were within normal limits. On further evaluation, the abdominal US showed a 3 cm × 4 cm appendicular phlegmon with minimal fluid leak. The patient was admitted for conservative management of a noncomplicated appendicular mass. Immediately, he was prescribed empiric broad-spectrum antibiotics including metronidazole and ceftriaxone.

**Figure 2:**
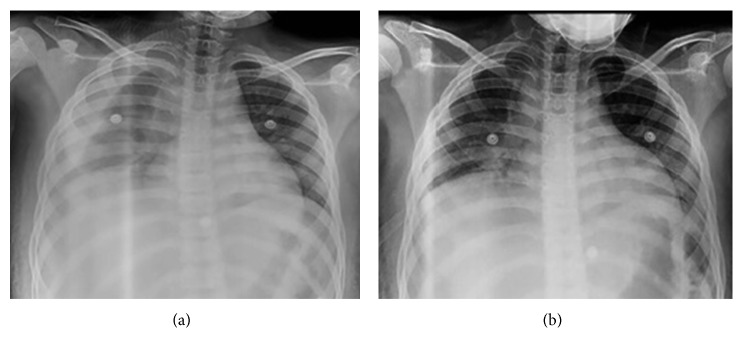
(a) Large right-sided pleural effusion and (b) evacuation of empyema after chest tube placement on the right side of the thorax.

**Figure 3:**
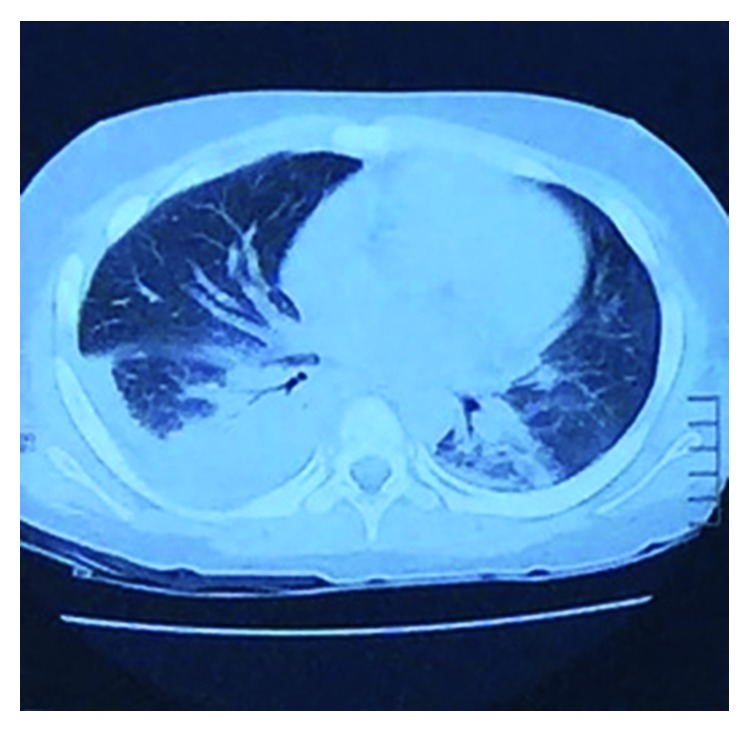
Axial view of the thoracic empyema. No underlying parenchymal disease was noted.

By day 3, the patient remained febrile. He developed severe respiratory distress, abdominal distention, and rebound tenderness. A computed tomography (CT) scan of the abdomen and pelvis revealed a secondary peritonitis for which an emergent open laparotomy was conducted. During the procedure, he was noted to have an appendicular abscess with 150 cc of pus and small collections throughout the pelvis. A few hours after surgery, the patient presented respiratory distress and hemodynamic decompensation. A chest X-ray revealed a large right-sided pleural effusion (Figure 2(a)). A chest and abdomen CT scan confirmed the effusion but failed to show evidence of parenchymal disease (Figure 3). A thoracentesis was conducted, and 200 cc of purulent liquid was evacuated and further analyzed.  Figure 2(b) shows the right thorax after thoracentesis and chest tube placement. Cultures from the pleural fluid were positive for extended-spectrum beta-lactamase *E. coli* (ESBL-*E. coli*) for which meropenem was started. During the following days, the patient improved his clinical status and progressively became afebrile. The chest tube was removed on day 14 after the resolution of empyema. Unfortunately, the patient was lost to follow-up.

### 2.2. Review of the Literature

We identified 10 cases of patients presenting with appendicitis and empyema (Table 1). The mean age was 25.1 years, and several cases were seen in children (60%). Furthermore, the male : female ratio was 0.5. Two distinctive case scenarios were identified: (1) appendicitis that is complicated with perioperative empyema and (2) empyema as the first clinical manifestation of appendicitis and the reason for consult. Seventy percent of the patients were either very young or old, for which premature or advanced age was considered a potential risk factor for this condition. Pregnancy could be another risk factor (10%). Enteric bacteria such as *E. coli, Bacteroides* spp., and *Klebsiella* spp. were the most common organisms isolated in the pleural fluid. All the patients survived and recovered. Initially, thoracic empyema was described in the USA in the early 1930s. However, recent cases have been described in both developed and developing countries (Spain, Argentina, etc.).

## 3. Discussion

In this report, we present a young patient who was admitted for conservative management of an appendicular mass. His clinical course was complicated with severe respiratory distress due to a thoracic empyema that was diagnosed postoperatively. This complication may appear despite a relatively early identification of the appendicular mass and prompt initiation of antibiotic therapy. A review of this topic including the pathophysiology of thoracic empyema, clinical scenarios, and potential outcomes is discussed.

Thoracic empyema may present in either young or elder individuals (2–68 years). However, pediatric patients are most frequently affected (60%), probably reflecting a difference in the pathological aspects of this condition between both groups. While in adults the omentum can contain the inflammation affecting the appendix, in children the omentum is underdeveloped and unable to limit purulent leakage [10, 11]. Thus, children are more prone to develop peritonitis compared to adult patients. Furthermore, children have a relatively mobile appendix compared to adults which may explain the increased frequency of nonfocal abdominal abscesses seen in this population [12].

Bacterial passage from the abdomen to the thorax is possible due to several mechanisms. For example, the pressure gradient between both compartments may cause a valve effect that favors the mobilization of intra-abdominal content to the thorax. Additionally, unilateral lymphatic flow is another factor described to play a role in the dissemination of pus across the diaphragm [4, 13]. Therefore, subhepatic, subdiaphragmatic, and more distant abscesses can spread to the thorax not only by contiguity but also by the anatomical and physical properties between these cavities [5, 14–16]. The migration of an appendicolith (dense mixture of stool and mineral deposit) from the abdomen to the thorax can also initiate an infection later in this compartment [9]. In some cases, it can be expelled following the perforation of the appendix and can form an abscess in the Morrison's pouch. Also, in exceptional cases, it can erode the diaphragm and predispose to purulent leakage to the thorax [9]. Moreover, pregnancy confers a special risk for thoracic empyema due to the relative immunosuppressive state associated to it and the displaced location of the cecum. For example, an appendiceal abscess in a pregnant woman may result in retroperitoneal collections that can gain access to the thorax through the retrocrural space and can result in extensive thoracic empyema [4].

Although our patient presented with obvious symptoms and signs of appendiceal phlegmon, it is possible that the previous use of oral nonsteroidal anti-inflammatory drugs prevented an early presentation to our ED. This situation posed the patient at an increased risk for complications such as abscess formation and peritonitis [17, 18]. Also, abdominal findings can vary depending on the severity of the disease and clinical scenario. For example, Wong and Kumar [7] and Tokat et al. [8] reported patients with marked peritoneal signs that were diagnostic for appendicitis but who developed empyema postoperatively. On the other hand, patients with ruptured appendix can present with an inconclusive abdominal evaluation and respiratory distress due to thoracic empyema [4, 5, 6, 15]. In the later scenario, respiratory distress can be the most prominent manifestation of the disease, and physicians may skip further investigation of the abdomen. Interestingly, the authors evaluated the abdomen as a source of infection only after ruling out an underlying lung disease.

Table 1 shows the most frequent organisms isolated in cases of thoracic empyema, including *E. coli*, *Bacteroides* spp., and *Klebsiella* spp. These organisms can tolerate significant oxygen concentrations and proliferate in the lung. According to the guidelines, appendiceal masses can be treated with antibiotic therapy when the mass is <5 cm, and there is no evidence of pus [19]. This is especially important as medical therapy may be as effective as percutaneous treatment (level of evidence IIB). In this report, our patient complicated his course with ruptured appendix, abscess formation, and infection of the pleural space despite the prompt use of broad-spectrum antibiotics. This is consistent with some studies that show that 19% of the patients with an appendiceal mass may fail to improve despite the appropriate use of antibiotics [19].

More invasive therapies such as early appendectomy are technically challenging, especially at the time of manipulating inflamed tissues or closing the appendiceal stump [20]. Therefore, the gold standard for the management of an appendiceal mass includes medical therapy with interval appendectomy. Nonetheless, those patients who fail to improve within the next 24–48 hours or who persist with fever or present worsening abdominal pain (worsening mass size) should undergo immediate surgery [21]. Our patient presented episodes of fever during the first 48 hours after starting antibiotics, which could be expected as the effect of antibiotics increase progressively. However, his worsening abdominal pain and respiratory distress during the third day of hospitalization was pathological and prompted emergent surgery with abscess removal.

Survival rate of patients with an appendiceal mass or abscess who develop thoracic empyema is 100%. This could be related to the aggressive surgical management and abscess washout once the patient begins to deteriorate [18, 22–23]. However, short- and long-term complications such as empyema necessitans, bronchopleural fistulas, and lung abscess have not been studied in this population and should not be overlooked. They may increase the risk of readmissions, prolonged hospital stay, and mortality rates, as reported elsewhere [24–26].

To the best of our knowledge, this is one of the few cases that present an appendiceal mass complicated with thoracic empyema. It can arise despite early identification and management of an appendiceal mass or abscess. Physicians should be aware of this complication during the perioperative state of appendicitis. Finally, the abdomen should be carefully evaluated when patients develop thoracic empyema without a clear parenchymal lung disease.

## Figures and Tables

**Table 1:** Cases of appendicitis associated with empyema.

Sex	Age	Risk factor	Isolated organism	Country	Outcome	Author
Male	5	Child	*Klebsiella pneumoniae*	USA	Survived	Stein et al. [22]
Female	50	—	*E. coli*, Enterobacteriaceae species	USA	Survived	Law et al. [5]
Male	5	Child	*E. coli*, *Streptococcus* spp., *Bacteroides fragilis*	USA	Survived	Law et al. [5]
Male	5	Child	*E. coli*, *Bacteroides* spp.	USA	Survived	Herline et al. [6]
Female	3	Child	*E. coli*, *Bacteroides ovatus*	Taiwan	Survived	Kao et al. [18]
Male	8	Child	None	New Zealand	Survived	Wong et al. [7]
Male	68	Elderly	*Klebsiella oxytoca*	Spain	Survived	García et al. [23]
Female	31	Pregnant	Gram-positive coccus	Argentina	Survived	Dietrich et al. [4]
Female	24	—	*E. coli*, *E. avium*	Turquía	Survived	Tokat et al. [8]
Female	2	Child	None	USA	Survived	Betancourt et al. [9]
